# OpenPore: A low-cost, portable, battery-powered exponential decay pulse generator for electroporation

**DOI:** 10.1016/j.ohx.2025.e00730

**Published:** 2025-12-14

**Authors:** Thomas Nesmith, Gagan D. Gupta, Darius G. Rackus

**Affiliations:** a Department of Chemistry and Biology, Toronto Metropolitan University, Toronto, Canada; b Institute for Biomedical Engineering, Science and Technology (iBEST), a partnership between St. Michael’s Hospital, a Site of Unity Health Toronto and Toronto Metropolitan University, Toronto, Ontario, Canada; c Keenan Research Centre for Biomedical Science at St. Michael’s Hospital, Toronto, Ontario, Canada; d ATEK Biomedical Systems Inc., Toronto, Ontario, Canada

**Keywords:** Electroporation, Pulse generator, Transfection, Mammalian cell

## Abstract

The OpenPore pulse generator is a battery powered, portable exponential decay pulse generator for performing mammalian cell electroporation. Electroporation is a common technique for transporting molecular cargo such as plasmid DNA through cell membranes. The system achieves this by providing a 0–330 V range with manual charge, pulse, and safe discharge controls as well as digital display indicating the stored voltage level. This is powered by two batteries (9 V and 1.5 V) allowing for a compact, lightweight system which can be implemented in a variety of settings based on experimental demands. When used in conjunction with a 0.4 cm commercial electroporation cuvette, transfection efficiencies comparable to commercially available systems are achieved.

## Graphical abstract

**Figure:**
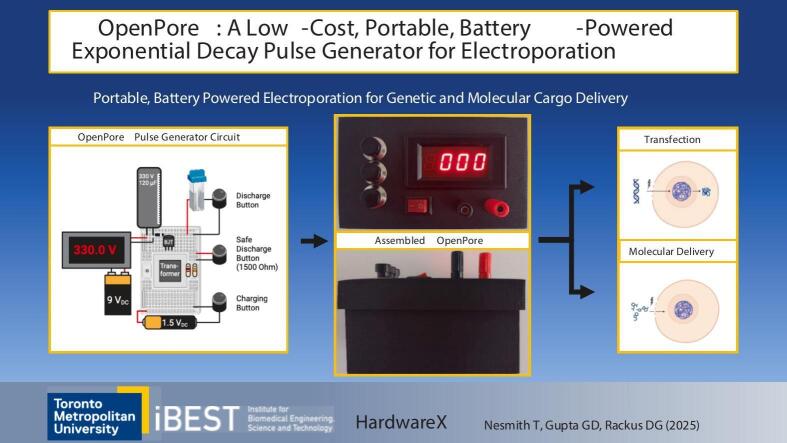

Specifications table

**Table:** 

Hardware name	OpenPore
Subject area	● Educational tools and open-source alternatives to existing infrastructure
Hardware type	● Biological sample handling and preparation
Closest commercial analog	Commercial analogues for electroporation include the Bio-Rad Gene Pulser and BTX Avantor. These instruments can provide similar exponential decay pulses in the same voltage range specifically for performing electroporation.
Open source license	Creative Commons Attribution 4.0 International Public License
Cost of hardware	The approximate cost of the device is $85 (CAD).
OSHWA certification UID *(OPTIONAL)*	UID CA000065
Source file repository	https://doi.org/10.17605/OSF.IO/642MV

## 1 Hardware in context

Electroporation is a common technique in biological research for transporting molecular cargo into various cell types [1]. This is performed using electric pulse generators which can provide specific high-voltage pulses, typically using square or exponential decay waves [2]. The technique can be further refined into reversible and irreversible poration, based on the ability of cells to recover membrane stability after poration. Reversible electroporation is commonly used in molecular cargo delivery into the cell. By temporarily producing pores in the cell membrane, genetic material can be transported into the cell without permanently destabilizing the cell membrane. In doing so, the cell can uptake the genetic material for expression while ensuring the viability of the cell. Consequently, electroporation has become a fundamental and routine technique in molecular and synthetic biology and is also finding application in medicine. These applications include electrochemotherapy and gene therapies for modifying tissues such as cancers as well as gene editing for treating genetic disorders. The primary advantages of these techniques include a drastic reduction in toxicity, high safety, and rapid delivery. [3], [4], [5], [6].

To support these techniques, many different instrument designs currently exist in the commercial space as well as custom designs for specific applications. Common commercial options include the Bio-Rad Gene Pulser, the ThermoFisher Neon Transfection, and Lonza Nucleofector. These instruments provide reliable bulk electroporation of resuspended cells such as yeast, bacteria and mammalian cells. Typically, this is achieved in parallel plate cuvettes, which are loaded with DNA and resuspended cells, followed by an electric pulse. Although their primary applications have been resuspended cells, multi-well cell culture plate electroporators are available for increased throughput [6]. Although these systems are effective, their size and financial burden are often prohibitive. The listed commercial options range in weight from approximately 6 to 8 kg, however this can increase when additional modules are installed, potentially doubling the weight, while occupying an approximately 30 × 30 cm footprint [7], [8], [9], [10].

Given the importance of electroporation in the fields of molecular, cell, and synthetic biology, many researchers have developed their own custom electroporation systems. Generally, these systems fall into two areas of development. The first group can be categorized as instruments aiming to replicate commercial performance but in a more lightweight or cost-effective design. These are often controlled by Arduino or similar microcontrollers to provide additional information to the operator such as operational state, pulse timing, or voltage level [11], [12], [13], [14]. The primary advantage of these systems is the drastic decrease in the cost of the system from thousands to hundreds of dollars (CAD). However, these systems often sacrifice functionality (typically waveform variety) to achieve a smaller, less complex system. Although some systems are portable as well, this is not a universal feature as many still require a standard wall outlet [15] or make use of high voltage batteries [11], [12], [13], [14]. Uniquely, the Electro-Pen sacrifices robust performance for extreme portability with a battery-free design, making use of the piezoelectric effect to produce high voltage exponential decay pulses. There is no pulse control, but the total cost of the device has been estimated to be under $0.53 USD [16].

The second area of development typically focuses on providing a specialized waveform for exploring novel biological responses such as bipolar, and nanosecond duration pulses [17]. Commercial options do exist for specific nanosecond pulse applications such as high-frequency irreversible electroporation (HFIRE). However, these are similarly bulky as well as prohibitive in cost [18]. There are similar devices that fit the portability and nanosecond pulse delivery requirements; however, they are still hundreds of dollars and require high-voltage power supplies [19], [20]. Others have begun adapting these techniques to small microscopy chambers for maximizing the available pulse voltage by reducing the chamber size [21]. This has led to advancements in miniaturization and the use of microfluidic chamber designs allowing for lower voltage and sample requirements with greater sample control [22]. However, these systems tend to have similar trade-offs, where portability is sacrificed for power requirements, or lack adaptability for continuous, live cell data collection.

Based on the limitations of current systems, we developed OpenPore, an exponential decay pulse generator, to provide a low-cost, portable, battery powered tool for electroporation. The combination of these features provides a flexible, mobile system which could be deployed in a variety of lab or clinical settings. With space already constrained in many facilities, it is advantageous to be able to implement experiments on-demand without bulky equipment. Its small platform and simple operation also make it well-suited for integration with custom platforms, such as microscopy controllers or advanced microfluidic control systems. Moreover, the low cost of the device allows for more researchers to access this technology and contributes to the growing demand for more precise techniques for modifying cells for research or clinical applications. Although currently this could assist many researchers, it can further expand access for training and education. Given the expensive nature of electroporators, few individuals can become proficient in these techniques, resulting in electroporation being vastly underutilized. By addressing these bottlenecks, advancements in electrochemotherapies as well as gene electrotransfer can be achieved faster, and novel treatments can be implemented sooner.

## 2 Hardware description

**Fig. 1:**
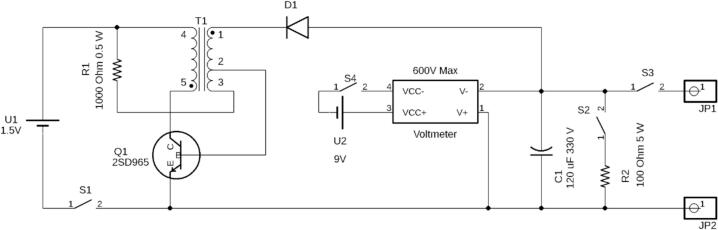
OpenPore exponential decay circuit diagram. The circuit diagram provides the intended assembly and implementation of the electronics. The circuit has two major sections to the design. On the far left of the circuit is the astable Joule Thief circuit for charging the primary capacitor (C1) triggered by S1. The feedback between the transistor and transformer create an oscillation at a high frequency. This converts the 1.5 V battery to over 450 V pulses. These pulses charge C1 and the total voltage is measured by the voltmeter. The pulse can then be delivered through the “Safe Discharge” resistor (S2 and R2) or delivered to the desired chamber through the external electrodes (JP1 and JP2). It is important to note that the pins shown for T1 are arranged based on the structural design of the coils, not the board level construction. This is meant to clarify the function of the coils with respect to each other and the transistor (Q1). Pin 4 and 5 are the primary coil of the transformer. Pin 1 is the start of the secondary coil which then terminates at a shared pin (Pin 2). Pin 2 is connected to a second feedback coil which then terminates at a dedicated “Feedback” pin (Pin 3). In the assembly of the circuit board, Pin 2 and 3 are swapped, indicated in Fig. S1.

The OpenPore pulse generator provides an exponential decay pulse up to a maximum of 330 V in a portable, battery powered system using a low-cost design. In doing so, this device provides experimental flexibility not provided by many commercial systems. This pulse generator provides the ability to perform electroporation and pulsed electric field (PEF) experiments for mammalian cells without being restricted to access to wall power. Moreover, the reduced size of the pulse generator frees space in otherwise crowded experimental environments. Although the OpenPore utilizes only one type of pulse, by focusing on a single waveform a smaller circuit was achievable. Fig. 1 provides a circuit diagram outlining the design of the internal electronics of the system. It is important to note the design of the transformer (T1) indicates the internal structure and exact relationship between the coils. For a description of the circuit board assembly refer to Fig. S1 (available with the source repository files). This design is based on a “Joule Thief” design which is based on the concept of using an astable circuit to create an AC signal from a DC source which is then transformed to a higher voltage. Traditionally, this circuit topology includes a toroid coupled inductor or flyback transformer (air gapped coupled inductor) in these designs, which is critical for the process of “stealing” greater amounts of energy from the battery. The key characteristic is the ability to store large amounts of magnetic energy which can be released as a high voltage pulse requiring fewer windings than a step-up transformer. In doing so, higher voltage AC pulses are produced as the transistor oscillates on and off. These pulses can then be used to intermittently turn on an active component such as an LED or to charge a capacitor. If this is repeated fast enough the total effect appears as if a constant voltage is being provided, when the signal is actually a very fast AC signal [23].

In the OpenPore circuit design, the Joule Thief circuit does not implement a true flyback transformer or toroid coupled inductor and it does not store any energy in the core. Instead, a high-turn ratio step-up Inverter Transformer with a shell-type core is used which operates like a traditional step-up or step-down transformer except it also includes a small “feedback” winding (Pin 3–2) which is an extension of the secondary coil (Pin 1–2). By using a high turn ratio, small voltages can be converted to high voltage, albeit with lower current. Shell-type cores have the primary and secondary windings wound around the same section of a central limb of the transformer. In doing so, the direction of magnetic flux in the primary and secondary are the same direction. Conversely, the “feedback” winding is shorted with the secondary coil but is wound in the opposite direction to the primary and secondary. Both the “feedback” and secondary coils share a common current because of sharing Pin 2, however, this results in opposing magnetic flux directions since they are wound in opposite directions. This characteristic is important to creating an oscillation between the transistor and the transformer. When the charging switch (S1) is closed, current can flow through both the base and collector of the transistor from the battery (1.5 V). The transistor turns on for two reasons. First, current in the feedback coil can now flow through the base turning on the transistor immediately as the switch closes. Second, as collector current flows, the primary coil begins to induce current in the secondary coil in the same direction as the current in the feedback coil. The combination opens the transistor leading to a high negative voltage at D1 which pulls positive charge from the negative terminal of the capacitor increasing the charge stored. This pulse is limited by the time it takes for the magnetic flux to increase high enough to lower the voltage at the base. Once this happens the collector and base current rapidly closes the transistor resulting in a voltage swing in the opposite in the primary, and secondary. This does not charge the capacitor given the placement and orientation of D1 but it does allow for the system to reset back to starting conditions, allowing the oscillation to continue. Ultimately, this can use a low-voltage power source (1.5 V) to create up to 330 V pulses capable of being stored on a capacitor for later discharge. This circuit is housed in a 3D printable chassis with dimensions measuring 14 cm × 8 cm × 6.5 cm. The limited parts and simple design allow for ease of assembly of the circuit as well as the final chassis. As PEF research expands, new treatments and techniques will be in greater demand, particularly for applications in clinical settings. By providing a low-cost, battery powered pulse generator, more facilities can gain access to these techniques.

Compared to non-commercial systems, the OpenPore focuses on battery power as a key feature. In doing so, this lowers the cost compared to systems implementing an external power supply. Additionally, this further reduces the size and portability of the system. Despite the focus on portability, several user features are provided above what is provided in the lowest cost devices such as the ElectroPen [16]. Operational features include chassis-mounted push buttons for pulse delivery, charging, and safe discharge of the pulse generator. Additionally, a voltmeter mounted on the front of the device allows for real-time monitoring of the voltage level of the pulse. This also provides an indication of the state of the pulse generator allowing the user to avoid accidental discharges or shock. This is powered by a second 9 V battery isolated from the charging circuit and can be independently turned on or off to conserve energy. This was done to ensure the voltmeter would have a stable power supply whereas the 1.5 V battery in the charging circuit is drained to charge the capacitor. In doing so, the capacitor can be safely monitored regardless of the state of the charging circuit battery.

1. OpenPore is a simple battery-powered handheld pulse generator for routine electroporation as well as customized experiments.
2. Suitable for mammalian cell transfection.
3. Compared to commercially available systems, the current system provides similar performance for drastically lower costs.
4. The system is small enough to operate within a biosafety cabinet or fume hood for added sample protection depending on experimental requirements.

## 3 Design files summary

**Table:** 

**Design file name**	**File type**	**Open-source license**	**Location of the file**
*OpenPore_Chassis_Design Files*	STL,DXF	CC-BY 4.0	https://doi.org/10.17605/OSF.IO/642MV
*OpenPore_CutPattern*	SVG	CC-BY 4.0	

## 4 Bill of materials summary

**Table:** 

**Designator**	**Component**	**Number**	**Cost per unit −currency**	**Total cost −** **currency**	**Source of materials**	**Material type**
S1, S2, S3	SW-PB1-1DZ-A-P1-A SPST Push Button (Normally “OFF”) 240 V, 3 A	3	$0.63 (CAD) + tax	$1.83 (CAD) + tax	https://www.digikey.ca/en/products/detail/adam-tech/SW-PB1-1DZ-A-P1-A/15284415	Composite
S4	SPDT Rocker Switch	1	$3.04 (CAD) + tax	$3.04 (CAD) + tax	https://www.digikey.ca/en/products/detail/e-switch/RB242C1021-114/3778090	Composite
T1	Inverter Transformer, 1.5 V to 300 V, 5 lead	1	$5.50 (CAD) + tax	$5.50 (CAD) + tax	https://theelectronicgoldmine.com/en-ca/collections/inverter-transformers/products/g23390	Metal
R1	1000 Ohm Resistor, 0.25 W	1	$0.16 (CAD) + tax	$0.16 (CAD) + tax	https://www.digikey.ca/en/products/detail/stackpole-electronics-inc/CF14JT1K00/1741314	Ceramic
R2	1500 Ohm, 5 W	1	$1.04 (CAD) + tax	$1.04 (CAD) + tax	https://www.digikey.ca/en/products/detail/te-connectivity-passive-product/EP5WS1K5J/8603501	Ceramic
Q1	NPN 2SD965	1	$0.10 (CAD) + tax	$0.10 (CAD) + tax	https://www.futurlec.com/Transistors/2SD965pr.shtml	Semi-conductor
D1	1 N4007	1	$0.15 (CAD) + tax	$0.15 (CAD) + tax	https://www.digikey.ca/en/products/detail/diotec-semiconductor/1N4007/18833652	Semi-conductor
U1	AA Battery (1.5 V)	1	$0.68 (CAD) + tax	$0.68 (CAD) + tax	https://www.digikey.ca/en/products/detail/panasonic-bsg/LR6XWA-BXU/2043737	Metal
U2	Battery (9 V)	1	$3.23 (CAD) + tax	$3.23 (CAD) + tax	https://www.digikey.ca/en/products/detail/zeus-battery-products/ZEUS-9V/9828849	Metal
C1	330 V, 120 μF	1	$9.96 (CAD) + tax	$9.96 (CAD) + tax	https://www.amazon.ca/-/fr/IBAPGXTTY/dp/B0CZQ26Y76	Metal
Voltmeter	Voltmeter Module (600 V Max) BERM 5135A	1	$18 (CAD) + tax	$18 (CAD) + tax	https://www.amazon.ca/Fielect-5135A-Digit-Digital-Voltmeter/dp/B08YDFNL2F?th=1	Composite
JP1, JP2	Banana Connector Jacks	2	$4.95 (CAD) + tax	$9.90 (CAD) + tax	https://sayal.com/STORE4/prodetails.php?SKU=154059	Composite
Protoboard (Used for assembling the circuit)	Protoboard	1	$4.95 (CAD) + tax	$4.95 (CAD) + tax	https://sayal.com/store4/prodetails.php?SKU=47468	Composite
9 V Battery Connector − Used with U2 in the circuit	9 V Battery Connector	1	$0.80 (CAD) + tax	$0.80 (CAD) + tax	https://www.digikey.ca/en/products/detail/keystone-electronics/233/68726	Composite
AA Battery Holder − Used with U1 in the circuit	AA (1.5 V) Battery Holder	1	$1.86 (CAD) + tax	$1.86 (CAD) + tax	https://www.digikey.ca/en/products/detail/keystone-electronics/2461/303810	Composite
Polylactic Acid (PLA) Black Filament – Used for 3D printing	Chassis and Holder (225 g + 85 g)310 g Total	1	$29.99 (CAD) + tax per 1 kg	$9.30 (CAD) + tax per 310 g	https://www.prusa3d.com/product/prusament-pla-jet-black-1kg/	Plastic
Insulated Wire – Used for connecting components on the circuit board as well as chassis mounted parts.	Jumper Kit Various 22AWG 70PCS	1	$8.06 (CAD) + tax per 70 pieces (multilength)	$3.00 (CAD) + tax per ∼ 25 wires. Estimate based on total connections used including potential losses during manufacturing.	https://www.digikey.ca/en/products/detail/global-specialties/WK-3/5231342	Metal
Stainless Steel Adhesive Tape 270 cm	Cuvette Holder Electrodes (L-Shape Strips)	1	$31.21 (CAD) + tax	$1.10 (CAD) + tax (10 cm)	https://www.amazon.ca/Silver-Temperature-Stainless-Acrylic-Adhesive/dp/B00L48YL0W	Metal

## 5 Build instructions

### 5.1 Build warnings

During construction it is dangerous to install the batteries, in particular the 1.5 V AA battery that powers the high voltage capacitor. Charging could be accidentally started if certain traces are bridged by direct contact with anything conductive. This could be metal tools or even bare skin if it is sufficiently damp. Therefore, it is necessary to not install the batteries until the circuit is fully complete and installed in the chassis. The circuit alone does not represent any danger if there is no power to it. However, caution should be taken when handling the circuit. Static discharge can be several thousands of volts and will damage the components. This can be prevented by wearing an anti-static wrist strap or regularly touching your hand to a metal object like a door handle. Use caution when operating a soldering iron. The tip can reach several hundred degrees and can cause serious burns. Consult with a trained individual on the proper use of a soldering iron before soldering is completed.

1. If using the 3D-printed chassis, use one of the model file types provided (SVG, STL, DXF) best suited to your equipment and begin printing in preparation for pulse generator assembly. This can take upwards of 24 h to complete and is best performed first. Depending on parts availability, this step can be used to modify dimensions for chassis mounted parts such as the voltmeter, switches, and output plugs. Depending on print quality and tolerance, some post-printing modification may be necessary to ensure a secure fit. Additional glues or epoxies may be used to provide additional support. Alternatively, a project box or plastic enclosure can be cut using the.SVG cutting pattern. After the chassis is printed the cuvette holder can be printed while constructing the pulse generator.
2. During chassis printing, the circuit board can be assembled using the circuit diagram provided and the protoboard (printed circuit board) as well as parts included in the Bill of Materials (BOM). It is recommended that traces are kept as short as possible to limit changes in the inductance of the circuit (Fig. 2). Fig. S**1** provides a parts placement diagram as an example arrangement of components and wires. The entire circuit can fit in space approximately 5 cm × 5 cm on the prototype board excluding the battery holders. Components may be substituted as needed, however pay close attention to the specifications for the transformer and transistor. For example, the more widely available XFT-5383 flyback transformer can be substituted for T1 and the appropriate pin configuration for this part is given in Fig. S**2**. These are specifically chosen to produce the astable circuit. The transistor must be able perform high frequency switching, while the transformer must be the correct winding configuration as well as having the correct turn ratio.

**Fig. 2:**
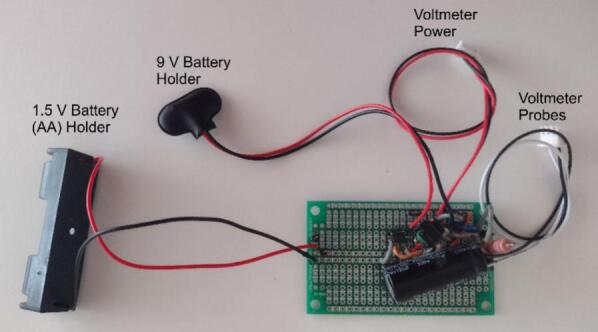
Assembled OpenPore exponential decay circuit. This photograph provides an overview of the assembled circuit board for the OpenPore exponential decay pulse generator. Indicated in the image are the commercially available battery holders (1.5 V and 9 V) as well as the voltmeter power and voltmeter probe cables provided with the 600 V voltmeter. Jumper wires are used to then permanently connect the circuit board to the switches on the chassis.
3. Once the circuit board is assembled and the chassis has been 3D-printed, final assembly can begin. Do not install the batteries prior to completing all electrical connections to avoid accidental charge or discharge of the capacitor. The system can produce high voltage during charging and can cause serious harm if exposed. Ensure all standard electrical safety practices are being followed. The capacitor should be manually discharged prior to maintenance or handling of the circuit. This can be performed using a high value resistor (>1500 Ohm) across the capacitor pins held by an insulated tool or a specific capacitor discharging tool can be used. Following this procedure will prevent sparks as well as excessive current flow which can reduce the lifespan of the capacitor. Discharge could take several seconds, and the user should re-test the capacitor to ensure it shows zero volts on a digital multimeter. This process is aided by the digital voltmeter installed in the system as well. Charge will slowly be lost to the voltmeter module, ensuring the system remains at zero volts when not actively being charged. It is recommended to leave the voltmeter installed on the high voltage board as an additional protective measure. All chassis-mounted parts can be installed, and connections can be made to the main circuit board. For a permanent connection, jumper wires should be soldered directly to the board ensuring secure connections to the chassis mount switches. Disconnections could cause permanent damage to the system and may compromise operator safety. The AA battery holder (U1), 9 V battery holder (U2) and circuit board may be secured using glue or epoxy for organizing the interior of the chassis. The standard assembly configuration for the system’s UI is shown in Fig. 3. It is important to not allow the high voltage circuit to contact the voltmeter on the upper chassis. If the high voltage board is secured to the bottom of the chassis, there is a substantial air gap that will insulate the two circuits.

**Fig. 3:**
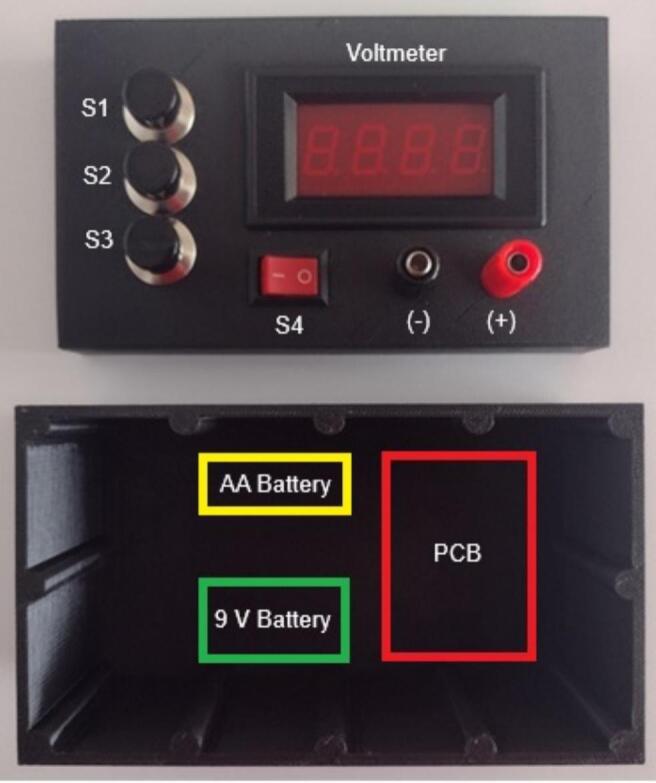
Unassembled OpenPore chassis. This picture displays the pre-assembled OpenPore 3D-printed upper and lower chassis parts based on the provided CAD file. The upper part provides the mounting points for the system switches, voltmeter and output terminals for pulse delivery. The lower part provides space for safely distancing all electrical components to prevent arcing between the circuit board and the upper chassis components. Additionally, this provides the user greater safety when replacing the batteries allowing for more space around the circuit board. Outlined in yellow (AA battery), green (9 V battery) and red are the preferred placement for the batteries and the printed circuit board (PCB). These can be secured using epoxy or hot glue. (For interpretation of the references to colour in this figure legend, the reader is referred to the web version of this article.)
4. After ensuring all connections are made between the circuit board and chassis mount parts, install both the 1.5 V AA battery and the 9 V battery (U1 and U2). At this point avoid touching any part of the circuit board until the batteries have been disconnected and the capacitor is discharged. Power on the voltmeter using S4 and ensure that the seven segment display illuminates. At this stage, the voltmeter can be calibrated using a small variable resistor on the rear of the voltmeter circuit board. This can be performed by connecting the capacitor to a Digital Multimeter (DMM) and charging the capacitor to a low voltage (∼50 V). Then read the DMM and voltmeter values. Adjust the resistor as necessary to match the values. For improved accuracy this can be repeated at multiple voltages. Again, ensure proper electrical safety practices are being followed to avoid accidental shock.
5. Finally, close the chassis by pressure fitting the upper lid to the lower chassis and the OpenPore is safe for regular operation. The completed system is shown below in Fig. 4. Do not permanently bond the two pieces as it is necessary to replace the batteries periodically. Based on the operational range of 150–250 V each AA battery should provide at least 500 pulses. Validation of the system did not exhaust the original AA battery with approximately 50–100 pulses. Measurement of the battery did not indicate any significant voltage drop after the resulting validation. Although thermal loss is likely occurring, it can be assumed the AA battery can provide several hundred pulses for mammalian cells. If the two chassis pieces do not fit snugly enough to prevent them from coming apart, tape, or rubber bands can be used for additional support, while ensuring access to the interior of the pulse generator. When not in use, turn off the voltmeter using S4 to prevent draining the 9 V battery unnecessarily.

**Fig. 4:**
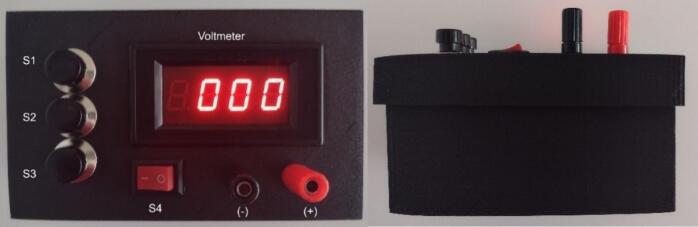
Assembled OpenPore chassis and circuit. Photographs of the top (left) and side (right) of the fully assembled OpenPore exponential decay pulse generator with the voltmeter powered, indicated by the lit numerical display of the voltmeter. Switches S1, S2, S3 are configurable for charge, discharge and safe discharge while switch S4 controls the power to the voltmeter. The standard configuration for S1-3 is setup with safe discharge at S3, while discharge is connected to S1. This ensures the operator does not reach over the discharge switch to render the pulse generator safe. The external output ports for the pulse generator are in the bottom right corner indicated by (−) and (+) for the respective polarities of the capacitor.
6. After the main pulse generator is complete, the cuvette holder can be rapidly assembled. The final design requires only two pieces of L-shaped adhesive backed stainless steel. These are placed as the contact electrodes for the cuvette and are folder around the cuvette holder as shown in Fig. 5. Alligator clip cables can then be looped through the appropriate guides on the holder to safely connect the holder to the pulse generator.

**Fig. 5:**
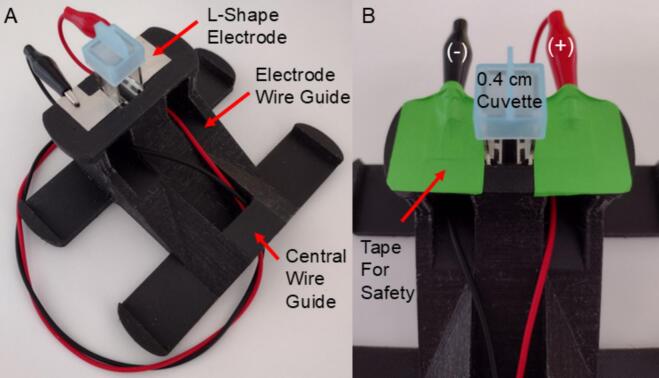
OpenPore 3D Printed Commercial Cuvette Holder. This image provides a view of A) the fully assembled OpenPore cuvette holder with corresponding alligator clip cables for connecting the pulse generator. B) Tape should be placed overtop the alligator clips for additional safety when operating the pulse generator. This will limit accidental contact between the operator and the cuvette electrodes.

## 6 Operation instructions

### 6.1 Operational safety warnings

This system can produce momentary pulses of high voltage as well as store up to 330 V in the capacitor. Caution should be used at all times when operating the OpenPore system. Ensure hands always remain free from the output electrodes and chamber to avoid accidental shock during pulse delivery. Do not operate the system with damp or wet hands as this can damage the system or result in accidental shock. Only clean the OpenPore system with 70 % ethanol which will quickly evaporate. Avoid exposing the circuit board to ethanol during cleaning. Corrosion can occur if exposed and not promptly wiped away. Ensure that the system is completely discharged when not in operation using the safe discharge feature. Never simultaneously charge and discharge the pulse generator. When simultaneously pressed, this can cause unpredictable voltage spikes inside the circuit which could damage the system.

1. First, place the OpenPore on a stable level surface close to where the pulse is going to be delivered. Banana plug to alligator clip electrodes or alligator clip to alligator clip cables can be used to provide a connection to the plate or cuvette being used. These can be commercial, or custom made depending on the application. Turn on the voltmeter using S4. The voltmeter should display 4 digits of a seven-segment display (or similar if an alternate display was installed).
2. Then connect the cuvette holder using alligator clips by passing them through wire guides as shown in Fig. 5. Once connected, the alligator clips and exposed electrodes on the holder should be taped over. This will help prevent sudden disconnections as well provide additional electrical safety for the operator. Once the pulse chamber of choice is safely connected to the pulse generator, the pulse generator is now ready for normal operation. The required voltage for transfection will vary and should be determined in advance of electroporation.
3. To charge the device, press and hold S2 while observing the voltmeter. This will happen quickly and can reach nearly 250 V in 10 s. While the theoretical maximum voltage is 330 V, it is recommended to operate below 250 V to ensure longevity and reliability. Once the desired voltage is reached on the voltmeter, release the push button. If the desired voltage is exceeded two options are available. First, the voltmeter will slowly draw current from the capacitor and therefore will slowly discharge over time. The operator may elect to wait for the voltage to discharge slightly before discharging the capacitor. The second option is to press and hold S2 which is meant to route the pulse through a 1500-ohm, 5-watt resistor, allowing for the capacitor to be safely discharged. The operator then may recharge the capacitor to the desired value.
4. When ready to perform the pulse, press and hold the pulse delivery switch (S3) until the voltmeter reads 0 V. If the capacitor only partially discharges, ensure the connection to the pulse chamber is properly connected. Alternatively, the chamber may have a high input impedance causing a low rate of discharge.
5. After successfully discharging the pulse, ensure the voltmeter reads 0 V before turning off the power to the voltmeter. If voltage shows, press S2 to discharge the remaining energy in the capacitor. Then disconnect the pulse generator electrodes from the chamber. Finally, turn off the voltmeter using S4 to ensure the 9 V battery is not drained unnecessarily.

## 7 Validation and characterization

The capabilities of the OpenPore are listed below:

● Mammalian cell electroporation
● Exponential decay pulse waveform only
● Monopolar pulses
● 0–330 V range
● Battery powered (9 V and 1.5 V)

**Fig. 6:**
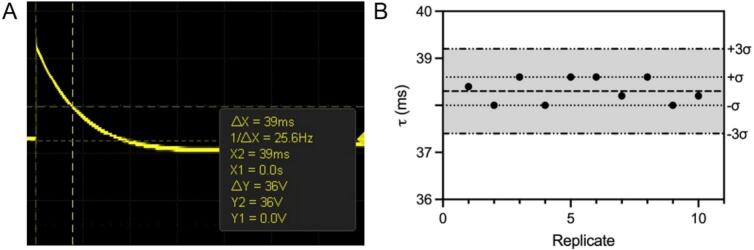
OpenPore pulse waveform validation measurements. Oscilloscope exports of the discharge waveform of the OpenPore set to 100 V delivered into a 0.4 cm Bio-Rad electroporation cuvette with a 100 μL of DMEM − High Glucose culture media. A) Graphical calculation of 1 τ which is the time when 63.2 % of the voltage is discharged. Given the precision of the oscilloscope, 64 % (or 64 V) was used to measure τ. B) Control chart for pulse replication. For 10 replicates, a mean τ (dashed line) of 38.3 ms was measured. The shaded region covers ± 3σ (dash-dotted and ± σ is indicated by the dotted line (±0.3 ms).

The performance of the pulse generator was verified by using an oscilloscope shown in Fig. S**3** and Fig. S**4.** In Fig. S**3** the transistor is measured at each of the terminals while in Fig. S**4** the transformer is measured at the secondary output as well as the base and collector of the transistor. Validation of the output signal from the pulse generator was performed and shown in Fig. 6 below. A) A 100 V test pulse was discharged into a 100 µL DMEM sample to record the resulting exponential decay pulse through the resistor. This allowed for the measurement of τ, which corresponds to the time at 62.3 % discharge. Precise measurement of the timing at that resolution was difficult so 64 % or 36 V was used to analyze the pulse which is slightly after 1 τ. The waveform showed an increase in the expected value of 12 ms (120 µF, ∼100 Ohm). The closed loop of the circuit between the capacitor and the load allows for slight oscillation into negative voltage before settling to zero volts. B) τ was then calculated using 10 consecutive 100 V pulses, which remains stable with a mean of 38.3 ms and with a standard deviation of 0.270 ms. Most of the variation in the average is likely due to measurement precision rather than actual variations in the electrical parameters over time.

### 7.1 Relevant use case: electroporation with commercial and custom microfluidic electroporation cells

The OpenPore was designed for performing exponential decay mammalian cell electroporation experiments given the operational voltage range. More robust cells with thicker outer membranes such as plants, yeast, and bacteria require more voltage.[24] This typically involves the transfer of plasmid DNA into a cell line for genetic modification known as gene electrotransfer (GET). Often these techniques are used in combination with microfluidic platforms, adding further experimental complexity. Future experimentation is expected to rely more heavily on microfluidics and by downsizing the sample volume [25]. Therefore, the relatively low power of the OpenPore system can be further leveraged for future experimental designs. To test the suitability of the OpenPore system, these methods were validated in two experimental assays. The first experiment was designed to test transfection efficiency against a benchmark provided by a Bio-Rad Gene Pulser. The second experiment tested interoperability of the OpenPore system with non-commercial equipment such as custom microfluidic chambers designed for low-volume sample handling.

### 7.2 Cell line and reagent preparation

Electroporation with a commercial electroporation cell was performed using 3 μg of Enhanced Green Fluorescent Protein (pEGFP) plasmid DNA (Addgene) per 100 μL cell culture. Plasmids were obtained from bacterial culture and isolation with a GeneAid plasmid DNA kit (GeneAid). The pEGFP obtained was stored in the provided GeneAid elution buffer until required for experimentation. U-2 OS osteosarcoma human cells (ATCC) were used and resuspended at approximately 6 × 10^6^ cells/mL in DMEM – High Glucose (Gibco). Both the DNA and cell culture used for the experiment were prepared as a master stock to ensure that both electroporation systems received identical ratios of cells to plasmid DNA.

Microfluidic electroporation validation was performed using YO-PRO™-1 (HelloBio), a cell-impermeant nucleic acid stain, as an indicator of pore formation. Prior to pulse delivery, YO-PRO™-1 was combined with 1 mL of resuspended U-2 OS cells at a concentration of 1 μM. In addition, Hoechst-33342 was added at a total concentration of 1 μM as a live cell stain allowing for efficiency analysis as well. Once completely dissolved, a 10 μL sample of resuspended cells and dyes was added to the microfluidic chamber. After the cells had settled to the floor of the chamber, a control image was taken to observe the initial number of permeabilized cells prior to pulse delivery. This provides an indication of the baseline cell death due to cell resuspension and trypsinization, which can be compared to the post pulse results. Immediately after the pulse, a second image was taken to observe the change in the number of fluorescent cells.

### 7.3 *Custom microfluidic* electroporation *chamber*

The custom microfluidic chamber was designed to emulate a conventional parallel plate configuration in combination with a linear microchannel allowing for reagent flow through. The channel was constructed by layering aluminum foil (Alcan 16 μm thick) between a polyethylene (Uline) upper layer and coverslip glass base (Sigma-Aldrich). Double-sided adhesive ARcare90106 (Adhesives Research, Inc., 58 μm thick) was used to seal each layer, enclosing the channel. This resulted in a channel measuring 0.132 mm × 1 mm × 20 mm with an internal volume of approximately 2.5 μL. Access to the channel was achieved by creating two 1 mm wide reagent ports: one at either end of the channel. Fig. S**5** in the shows a cross-section of the chamber, outlining the layering used for constructing the chamber.

### 7.4 Electroporation

For the comparison with a commercial system, GET was performed with both the Bio-Rad Gene Pulser and the OpenPore in triplicate over a range of five voltages (160, 180, 200, 220, and 240 V). The capacitance on the Gene Pulser was set to 125 μF, which is the closest to the OpenPore’s fixed 120 μF capacitance. Each 100 μL cell sample was taken from a master stock prepared on the day of the experiment to ensure both systems received identical ratios of plasmid DNA to cells. Each replicate was electroporated and plated in a 12-well plate followed by 24-hour incubation. After 24 h, the cells were fixed using 4 % paraformaldehyde (PFA) in phosphate buffered saline (PBS) and stained using 1 mM 4′,6-diamidino-2-phenylindole (DAPI) in PBS. This was performed by first washing the samples with PBS (1 mL). Then 4 % PFA (0.5 mL) was applied to the sample and incubated for 10 min to ensure complete fixation. Afterward the sample was washed again using 1 mL of PBS and then 0.5 mL of 1 mM DAPI was applied and the samples were incubated for another 10 min. Finally, the cells were washed again with 1 mL of PBS and then stored with 1 mL of PBS before imaging.

For the demonstration of electroporation with a custom microfluidic electroporation cell, a single 40 V pulse (400 V/cm) was delivered to a 2.5 μL volume of resuspended U2 OS cells.

### 7.5 Microscopy and electroporation efficiency determination

Results of both electroporation experiments were observed using a Delta Vision fluorescent widefield microscope at 20 × objective magnification. The FITC filter set (Ex: 498 nm, Em: 517 nm) was selected for the pEGFP construct and the DAPI filter set (Ex: 359 nm, Em: 461 nm) was selected to detect nuclear staining. Transfection efficiency was determined by selecting 6 images per sample using the DAPI filter only which maximized the number of individual cells in each image at each voltage. This provided a consistent baseline indication of cell viability after electroporation as evidenced in Fig. S5. The low standard error shows that each of the 6 images were close in cell density for each of the 3 samples. This also provided a way to blindly sample GFP while ensuring the highest possible number of countable cells. By not viewing GFP signal during cell selection the positive counts were more random as indicated in the standard error shown in Fig. S5. These were identified manually by looking for co-occurrence of DAPI and GFP signal as well as healthy cell morphology. Cells that had co-occurrence, but improper morphology (i.e. apoptotic, or necrotic) were excluded from the data. Cells cut off at the image edge were also excluded from the counts since morphology could not be verified. Positive signal (DAPI and EGFP) was determined as being several times the intensity of the background signal in both channels. This cell counts provided an indication of cell viability and average transfection rates. The average transfection rate was determined across the ranges from 160 to 240 V.

## 8 Results

**Fig. 7:**
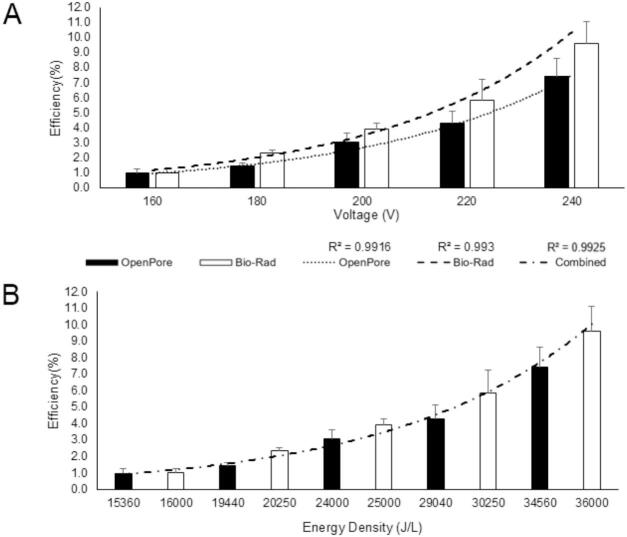
Average Transfection Efficiency Compared Between OpenPore and Bio-Rad Electroporation Systems. A) Transfection efficiency as a function of applied voltage for the OpenPore (black) and Bio-Rad Gene Pulser (white) electroporation pulse generators. Transfection efficiency was determined using 100 μL of resuspended U2 OS cells and 5 μg of EGFP plasmid DNA in triplicate for 5 voltages ranging from 160 V to 240 V. N = 3, error bars = ± 1 standard. B) Transfection efficiency as a function of energy density across for both the OpenPore and Gene Pulser. This is a combination of the two graphs in A above, adjusting for the small variation in capacitance of the systems which changes the duration of the pulse. N = 3, error bars =± 1 standard deviation.

Electroporation efficiency is the ratio of cells that have been successfully transfected to the total number of viable cells after plating. Electroporation efficiency as a function of voltage for both the OpenPore and the Bio-Rad Gene Pulser were compared across a range of voltages and are presented in Fig. 7. (Raw cell counts are provided in Fig. S**6**). On a pulse voltage basis (Fig. 7A), the OpenPore pulse generator had comparable electroporation efficiencies, with slightly lower efficiencies obtained by the OpenPore. This slight discrepancy is likely caused by the difference in capacitance between the two systems. The larger capacitance of the Gene Pulser, results in a longer theoretical pulse time, increasing the power delivered by the pulse. When the electroporation efficiencies are instead plotted as a function of energy density (Fig. 7B) and compared, the data from the OpenPore and the Gene Pulser are derived from the same correlation. This indicates that both systems were operating as expected, achieving the appropriate transfection efficiency independent of pulse length.

**Fig. 8:**
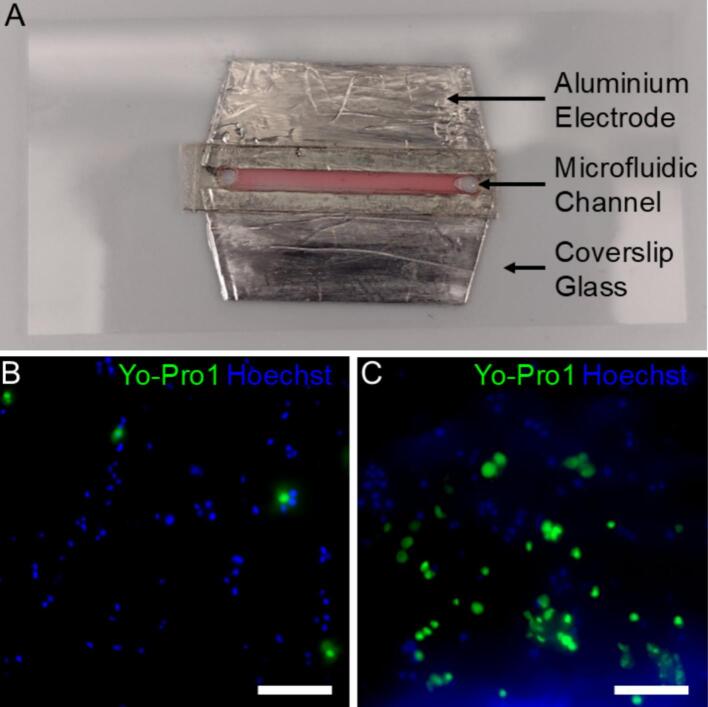
Microfluidic electroporation validation using U2 OS cells and YO-PRO™-1. (A) Photograph of assembled custom microfluidic electroporation chamber. The chamber comprises a pair of aluminum electrodes 20 mm in length and spaced 1 mm apart. The coverslip glass measures 50 mm by 25 mm and can be fitted to standard microscope stages and environmental chambers. The microfluidic channel holds approximately 2.5 µL of cell resuspended cell culture. The channel was mounted on cover slip glass, allowing for higher resolution microscopy. (B) & (C) Composite fluorescence micrographs of adherent U-2 OS cells within the microfluidic electroporation chamber in the presence of YO-PRO™-1 before and after electroporation, respectively. Scale = 50 µm.

The simplicity of the OpenPore makes it ideal for integration with custom systems as well beyond routine GET procedures. To demonstrate the OpenPore’s ability to operate with non-standard and custom electroporation configurations, it was combined with a custom microfluidic electroporation chamber (Fig. 8A). The 0.1 cm electrode gap and 40 V pulse resulted in an electric field strength of 400 V/cm, which was equivalent to a pulse of 160 V using a commercial electroporation cuvette with 0.4 cm electrode spacing. Here, electroporation enabled delivery of the cell-impermeant dye YO-PRO™-1. Fluorescence microscopy images obtained before and after electroporation in the presence of the dye are given in Fig. 8B**&C**. Post-electroporation, there was a visible increase in YO-PRO™-1, indicating successful permeabilization. Moreover, by using the custom microfluidic electroporation chamber, electroporation was achieved with 4 × lower voltage, increasing the longevity of the battery used for pulse generation. This is highly advantageous, demonstrating the benefits of combining the OpenPore electroporator with microfluidic platforms.

The OpenPore pulse generator provides accessible electroporation in a mobile platform. By implementing battery power, the pulse generator can be used on-demand in a compact package. This enables it to be implemented in a variety of scenarios such as a within a biosafety cabinet or fume hood, on a bench top, and with microscopy, or a combination of these in one experiment. The lack of mains-source power being provides greater freedom to experiment as well as a reduction in setup and teardown times. As electroporation becomes more popular, throughput and ease of operation will be greatly important for researchers and lab technicians. We also expect the OpenPore to be of use to citizen scientists and those involved in grass-roots synthetic biology communities like iGEM.

Given the reliability of the platform, there is potential for further advancement in the design providing additional features. Electrochemotherapy and electroporation for gene therapy are growing in clinical application. While not specifically designed for clinical applications, portable instrumentation like the OpenPore will be beneficial for such treatments. Moreover, the flexibility to pair with custom chambers provides researchers with the ability to execute more types of experiments as well as lift restrictions on future designs requiring specialized electrical connections.

## CRediT authorship contribution statement

**Thomas Nesmith:** Writing – original draft, Visualization, Validation, Resources, Methodology, Investigation, Formal analysis, Conceptualization. **Gagan D. Gupta:** . **Darius G. Rackus:** Writing – review & editing, Supervision, Resources, Funding acquisition.

## Declaration of competing interest

The authors declare the following financial interests/personal relationships which may be considered as potential competing interests: **Thomas Nesmith** is the founder and owner of ATEK Biomedical Systems Inc., which provided matching funding for a Mitacs Accelerate grant. **Darius G. Rackus** is a member of the Scientific Advisory Board for World Precision Instruments, Inc., for which he receives compensation. **Gagan D. Gupta** has no conflicts to disclose.
